# A comprehensive evaluation of single nucleotide polymorphisms associated with atrophic gastritis risk: A protocol for systematic review and network meta-analysis

**DOI:** 10.1097/MD.0000000000020677

**Published:** 2020-07-17

**Authors:** Zhen-Yu Tang, Zhuo-Miao Ye, Jing-Hui Zheng, Feng Jiang, You-Ming Tang

**Affiliations:** a Guangxi University of Chinese Medicine; b Ruikang School of Clinical Medicine, Guangxi University of Chinese Medicine; c Department of Cardiology, Ruikang Hospital Affiliated to Guangxi University of Chinese Medicine; d Department of Gastroenterology, Ruikang Hospital Affiliated to Guangxi University of Chinese Medicine, Nanning 530011, China.

**Keywords:** atrophic gastritis, network meta-analysis, SNPs, susceptibility

## Abstract

**Background:**

Single nucleotide polymorphisms (SNPs) have been inconsistently associated with atrophic gastritis (AG) risk. This meta-analysis aimed to synthesize relevant data on SNPs associated with AG.

**Methods:**

To identify all associated studies of SNPs and AG published, databases had been searched through January 2020 from the databases of PubMed, China National Knowledge Infrastructure (CNKI), Web of Science, Embase, the Chinese Science and Technology Periodical Database (VIP), Cochrane Library, and Wanfang databases. With the help of network meta-analysis and Thakkinstian algorithm, the best genetic model with the strongest correlation with AG was selected, the final result – matching to the noteworthy correlation – was obtained by referring to the false positive reporting rate (false positive report probability, FPRP). Based on STREGA's stated criteria, the methodological quality of the data we collected was valued. Both Stata 14.0 and GeMTC will be used for a comprehensive review of the system and will be used in our meta-analysis.

**Results:**

This study will provide a high-quality evidence to find the SNP most associated with AG susceptibility and the best genetic model.

**Conclusions:**

This study will explore which SNP is most associated with AG susceptibility.

**Registration:**

INPLASY202050016.

## Abstract 2

Supplemental Digital Content is available in the text

## 1 Introduction

Chronic atrophic gastritis (CAG) is a chronic disease of digestive system caused by multiple pathogenic factors, often accompanied by intestinal metaplasia and dysplasia, which is considered as a precancerous disease of the stomach. The annual incidence of gastric cancer (GC) in CAG patients within 5 years after diagnosis was 0.1%.^[1]^ CAG is easy to turn into GC. The annual incidence of GC in CAG patients is 0.2% in China, 0.1–1.1% in the UK, 0.2% in Japan, and 0.2% in Italy.^[2]^ CAG, due to the invasion of inflammation, eventually leads to the loss of mucosal glands. The tissue changes may be due to the autoimmune mediated response, which is also related to the infection with helicobacter pylori.^[3]^ In addition, the risk factors of CAG are related to age,^[4]^ mental factors,^[5]^ BMI,^[6]^ etc. Single nucleotide polymorphisms (SNPS), the most common form of gene variation in the human genome, have a certain influence on cancer susceptibility.^[7,8]^ With the deepening of modern studies on the correlation between atrophic gastritis (AG) and SNP, we have found that PTPN11,^[9]^ IL-1B,^[10]^ PRKCH,^[11]^ PSCA,^[12]^ and other SNPS are related to AG, but these studies have a small sample size and inconsistent conclusions. Few studies have comprehensively summarized and evaluated all GC-related SNPs, so the purpose of this study is to comprehensively evaluate significant SNPs related to AG susceptibility. Currently, there is a lack of evidence to show which genetic model is best suited to identify the relationship between SNP and AG. Therefore, we used methods such as mesh meta-analysis to select the most suitable SNP and its genetic model for evaluating AG susceptibility

## 2 Methods

### 2.1 Registration

Our study has been registered on International Platform of Registered Systematic Review and Meta-analysis Protocols (INPLASY). The registration number was INPLASY202050016. More details can be seen in https://inplasy.com/inplasy-2020-5-0016/.

### 2.2 Ethics and dissemination

Since this study is a meta-analysis based on previously published literature, ethical approval and informed consent are not required and will be published in a peer-reviewed journal.

### 2.3 Eligibility criteria

**Participant or population:** Patients with AG.
**Intervention:** Gene polymorphism.
**Comparator:** People without AG.
**Study designs to be included:** Case-control studies.
**Type of outcomes:** AG risk comparisons.

### 2.4 Information sources and search strategy

Studies published through January 2020 that compared frequency differences in SNPs between AG patients and healthy controls were identified from PubMed, Web of Science, Embase, Cochrane Library, China National Knowledge Infrastructure (CNKI), the Chinese Science and Technology Periodical Database (VIP), and Wanfang databases, with no language limits. The search strategy was based on the following search terms: “single nucleotide polymorphism”, “SNP”, “atrophic gastritis”, and “chronic atrophic gastritis”. Details regarding the search terms are available in the Supplementary Materials S1.

### 2.5 Inclusion criteria

Case-control study, published in either English or Chinese that concern the susceptibility of the SNPs to the AG, will be incorporated in our review. No limitations of publication status or data will be settled. Studies reported in full-text will be screened for inclusion. The references of all eligible studies were manually screened to ensure that all relevant studies were included. Studies were considered only if the studied population who were taken serum samples before prior chemoradiotherapy and cancer risk was the outcome. No restrictions were placed on age, gender, country, or tumor stage.

### 2.6 Exclusion criteria

A study was excluded if it was a repeat report, conference report, thesis, review paper, or animal study, or had insufficient data for genotyping distribution calculation. Studies in which SNPs demonstrated a departure from Hardy–Weinberg equilibrium (HWE) in controls were excluded.

### 2.7 Data collection and analysis

Two reviewers conducted the selection process independently, with cases of disagreement resolved by discussion or consulting a third reviewer. Data extracted from each paper included: author, country of publication, year, number of men and women, sample size, race, and details of target SNPs, including genotyping methods, genotype frequency, and HWE values. For controls of each study, HWE was estimated using the goodness-of-fit test. For pairwise meta-analysis, a fixed- or random-effects pooled odds ratio (OR) with 95% confidence intervals (CIs) were calculated, depending on degree of heterogeneity under six genetic models (allele contrast model, homozygous model, heterozygous model, dominant model, recessive model, and over-dominant model). Heterogeneity was quantified with the *I*^2^ statistic and *P* value; a *I*^2^ statistic < 50% and a *P* > .1 indicated low heterogeneity between studies, in which case the fixed-effect model was employed. For significant SNPs with evidence of heterogeneity in meta-analysis, assessment of sources of heterogeneity was employed using subgroup analysis if sufficient data existed. Publication bias was assessed using the Begg and Egger tests.

A random-effects network meta-analysis within a Bayesian framework was conducted using the GeMTC software (v 0.14.3).^[13]^ Four parallel Markov chain Monte Carlo simulations were run for a 20,000-stimulation burn-in phase and an additional 50,000-stimulation phase. Convergence was satisfied with a potential scale reduction factor (PSRF) value of 1.0 as the cut-off value. Consistency, referring to agreement between direct and indirect comparisons in terms of effect estimates, was evaluated by comparing consistency model with inconsistency model in terms of standard deviation of the random effect. The inconsistency model was used when an obvious deviation was detected; otherwise, the consistency model was used. This Bayesian approach was used to rank the probability of each genetic model for risk assessment for AG and corresponding rank probability plots were generated.

We further compared genetic models to select the most appropriate model using the algorithm by Thakkinstian et al.^[14]^ To assess the noteworthiness of the normally significant SNPs under the most appropriate genetic model determined by network meta-analysis or Thakkinstian algorithm, FPRP was calculated assuming three levels of prior probabilities (low: 0.1; moderate: 0.01; high: 0.001) and an OR of 1.5, as previously described.^[15,16]^ Significant SNPs with a FPRP value < 0.2 were considered noteworthy.^[16]^ Diagnostic meta-analysis was conducted to determine sensitivity and specificity of SNPs in predicting AG risk using the Meta-DiSc software.^[17]^

### 2.8 Quality assessment

The methodological quality of data was assessed based on the STREGA statement.^[18]^ Two reviewers conducted the rating independently and a third reviewer was consulted for consensus if disagreement occurred.

### 2.9 Subgroup analysis and sensibility analysis

Subgroup analyses, which are designed for patients’ race, age, gender, the quality of literature, will be used to find the possible sources on account of a possibility of significant heterogeneity or inconsistency. Sensitivity analysis will be conducted to check the robustness and reliability of pooled outcome results.

### 2.10 Reporting bias

Reporting bias may affect the results of systematic reviews. The control of publication bias is more difficult and has a greater degree of impact. Therefore, the identification and processing of publication bias is an important step in systematic reviews. The funnel plot, a kind of visualization method, will be used to identify Reporting bias.

## 3 Discussion

This meta-analysis will evaluate and analyze the most appropriate SNPS associated with AG and their genetic models, and provide more evidence-based guidance for clinical treatment. In order to make more suitable studies concluded, we have searched several well-known international databases and commonly used databases in China. And we have developed suitable plans to deal with the risk bias, reporting bias and heterogeneity that may occur in this study. It should be noted that the present study may have potential limitations of homogeneity as a result of the various race. And our meta-analysis may need additional large sample size, detailed AG risk factor data and high-quality studies to explore the susceptibility between SNPs and the risk of AG. We believe that this systematic review will find the SNP most associated with AG susceptibility and select the most appropriate genetic models.

## Author contributions

**Analysis planning:** Jing-Hui Zheng, Zhuo-Miao Ye, Zhen-Yu Tang

**Conceptualization:** Jing-Hui Zheng, Zhuo-Miao Ye, Zhen-Yu Tang

**Data curation:** Zhuo-Miao Ye

**Draft manuscript:** Zhuo-Miao Ye

**Investigation:** You-Ming Tang

**Manuscript editing:** Zhuo-Miao Ye, Jing-Hui Zheng

**Methodology:** Zhuo-Miao Ye, Jing-Hui Zheng

## Supplementary Material

Supplemental Digital Content
