# Supplementary material for: A comprehensive evaluation of single nucleotide polymorphisms associated with atrophic gastritis risk: A protocol for systematic review and network meta-analysis
Source: Medicine (Baltimore). 2020 Jul 17;99(29):e20677. doi: 10.1097/MD.0000000000020677 (PMC7373582; doi:10.1097/MD.0000000000020677)
Supplement: Supplemental Digital Content [file medi-99-e20677-s001.docx]

Search ((((((((Nucleotide Polymorphism, Single[Title/Abstract]) OR Nucleotide Polymorphisms, Single[Title/Abstract]) OR Polymorphisms, Single Nucleotide[Title/Abstract]) OR Single Nucleotide Polymorphisms[Title/Abstract]) OR SNPs[Title/Abstract]) OR Single Nucleotide Polymorphism[Title/Abstract]) AND Humans[Mesh])) AND ((((Atrophic Gastritides[Title/Abstract]) OR Atrophic Gastritis[Title/Abstract]) OR Gastritides, Atrophic[Title/Abstract]) AND Humans[Mesh]) Filters: Humans
